# Role of Translocator 18 KDa Ligands in the Activation of Leukotriene B4 Activated G-Protein Coupled Receptor and Toll Like Receptor-4 Pathways in Neutrophils

**DOI:** 10.3389/fphar.2017.00766

**Published:** 2017-10-27

**Authors:** Léonard de Vinci Kanda Kupa, Carine C. Drewes, Eric D. Barioni, Camila L. Neves, Sandra Coccuzzo Sampaio, Sandra H. P. Farsky

**Affiliations:** ^1^Department of Clinical and Toxicological Analyses, School of Pharmaceutical Sciences, University of São Paulo, São Paulo, Brazil; ^2^Laboratory of Pathophysiology, Institute Butantan, São Paulo, Brazil

**Keywords:** neutrophils chemotaxis, TSPO modulation, LPS, LTB4, cofilin, Arp2/3, cytokines

## Abstract

TSPO (Translocator 18 KDa; tryptophan-rich sensory protein oxygen sensor) is a constitutive outer mitochondrial membrane protein overexpressed in inflammatory cells during local or systemic processes. Despite its expression is characterized, role of TSPO in inflammation remains elusive. For this study, we investigated the role of TSPO ligands on neutrophil functions elicited by two different inflammatory pathways. Peritoneal neutrophils were isolated from male Balb-C mice, treated with TSPO ligand diazepam, Ro5-4864 or PK11195 (1,100 or 1000 nM; 2 h) and further stimulated with lipopolysaccharide from *Escherichia coli* (LPS), a binding for Toll-Like Receptor-4 (TLR4), or leukotriene B4 (LTB4), a G-protein coupled receptor (GPCR) ligand. LPS treatment did not lead to overexpression of TSPO on neutrophils, and pre-treatment with any TSPO ligand did not alter cytokine expression, adhesion molecule expression, or the production of reactive oxygen and nitrogen species caused by LPS stimulation. Conversely, all TSPO ligands impaired LTB4’s actions, as visualized by reductions in L-selectin shedding, β2 integrin overexpression, neutrophil chemotaxis, and actin filament assembly. TSPO ligands showed distinct intracellular effects on LTB4-induced neutrophil locomotion, with diazepam enhancing cofilin but not modifying Arp2/3 expression, and Ro5-4864 and PK11195 reducing both cofilin and Arp2/3 expression. Taken together, our data exclude a direct role of TSPO ligands in TLR4-elicited pathways, and indicate that TSPO activation inhibits GPCR inflammatory pathways in neutrophils, with a relevant role in neutrophil influx into inflammatory sites.

**GRAPHICAL ABSTRACT 1:**
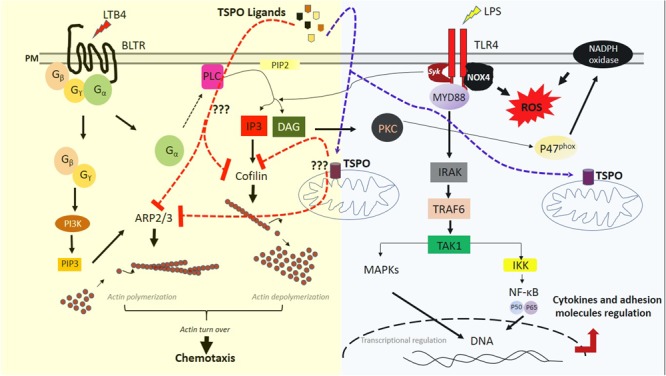
Proposal mechanisms of TSPO ligands on GPCR and TLR-4 pathways in neutrophils. In the left: GPCR (G-protein coupled receptor) pathway regulation by TSPO ligands. Upon BLTR (Leukotriene B receptor) activation by LTB4 (leukotriene B4), the coupled G-protein dissociates in two subunits. The Gβγ subunit activates PI3K (phosphatidylinositol-3-kinase) which leads to an intracellular increase of PIP3 (Phosphatidylinositol-3,4,5-trisphosphate). The increase of PIP3 is associated with activation of ARP2/3 (Actin related proteins 2/3), which mediates actin polymerization. In the other hand, the G protein subunit Gα activates PLC (phospholipase C) close to the plasmatic membrane (PM). PLC hydrolyses the membrane PIP2 (Phosphatidylinositol-4,5-bisphosphate) to form IP3 (Inositol triphosphate) and DAG (diacylglycerol). IP3 leads to cofilin activation, while DAG recruits protein kinase C (PKC), which activates p47phox, a subunits of NADPH-oxidase allowing NADPH-oxidase activation and reactive oxygen species (ROS) production. Cofilin activation leads to actin depolymerization and ensures, together with actin polymerization, the actin turn over and chemotaxis. TSPO ligands inhibit both cofilin and ARP2/3 expression, impairing chemotaxis. In the right: TLR4 (Toll like receptor) pathway regulation by TSPO ligands. LPS (lipopolysaccharides) activates TLR4, which is coupled with Syk (Spleen tyrosine kinase), NOX4 (NADPH-oxidase – 4) and the Myeloid differentiation primary-response protein (MYD88). NOX4 act as a direct ROS producer while Syk acts amplifying PLC pathway by increasing intracellular DAG and ROS production via NADPH-oxidase activation. Upon TLR4 activation, MYD88 recruits interleukin receptor-associated kinase proteins (IRAKs) which activates TRAF6(TNF Receptor-associated factor 6) which in turn activates TAK1(TGFβ activated kinase). TAK1 activation leads to IKK (Inhibitor of NF-κB kinase) complex activation and to MAPKs (Mitogen activated proteins kinase) activation. Activated IKK complex leads to NF-κB (Nuclear factor kB) activation and release while MAPKs activation leads to phosphorylation of several transcriptional factors. Both NF-κB and phosphorylated transcriptional factors migrate to the nucleus for transcriptional regulation. Then inflammatory mediators such as cytokines and adhesion molecules are regulated. TSPO ligands did not show any regulation in this pathway.

## Introduction

TSPO (Translocator 18KDa; tryptophan-rich sensory protein oxygen sensor) is mainly found as an outer mitochondrial membrane protein; it is required for the translocation of cholesterol, and thus regulates the rate of steroid synthesis (Selvaraj and Tu, 2016). TSPO was initially identified in the 1970s as a peripheral receptor to the benzodiazepines (PBR); nowadays, TSPO is known to be widely expressed throughout the body, including in the cells of the central nervous system (CNS), where it is considered a marker of neuroinflammation in systemic or local inflammatory diseases (Sandiego et al., 2015; Notter et al., 2017). Moreover, TSPO is overexpressed by macrophages at inflammatory and cancer sites (Hatori et al., 2012, 2015; Li et al., 2016; Zinnhardt et al., 2017), as well as by neutrophils that migrate into the liver after injection with cycloheximide (Hatori et al., 2014) or into the lungs after systemic injection of lipopolysaccharides (LPS) (Hatori et al., 2012).

Notwithstanding the knowledge that TSPO is overexpressed in stress conditions, the precise mechanism of TSPO in disease-related processes remains elusive (Selvaraj and Tu, 2016). Understanding the structure of the receptor and the sites of ligand binding are pivotal to uncovering the real effects of TSPO in pathophysiological scenarios (Li et al., 2015). TSPO is a dimer composed of five transmembrane helices, and the central cavity houses the binding sites for endogenous and drug ligands. The binding of ligands to different units in the core can be responsible for agonist or antagonist effects, depending on cell phenotype, and chemical class and concentration of ligand. A series of highly specific synthetic ligands for TSPO have been successfully developed as radiotracers for cancer and inflammation, and the mechanisms of action of two prototypical TSPO ligands, chlorodiazepam Ro5-4864 and isoquinolone carboxamide PK11195, have been widely investigated. Findings on Ro5-4864 and PK11195-binding responses, however, have proven inconsistent over the years, with effects ranging from steroid hormone production to cell signaling in TSPO-related diseases (Le Fur et al., 1983; Shany et al., 1994; Choi et al., 2011; Gatliff and Campanella, 2016).

Although TSPO is highly expressed on cells with innate immunity (Hatori et al., 2012, 2014; Cuhlmann et al., 2014; Sandiego et al., 2015; Notter et al., 2017), its role in neutrophils has not been fully investigated. Neutrophils are the most highly expressed leukocyte in human blood, and represent the first line of defense to infection. Circulating neutrophils rapidly respond to inflammatory chemical mediators or bacterial components by adhering to the vessel wall, migrating into the inflamed tissue, phagocytosing, and killing pathogens, and producing and releasing reactive oxygen (ROS), nitrogen species (RNS), cytokines, eicosanoids, proteases, peroxidases and extracellular traps (Deniset et al., 2016). Furthermore, recent data have identified additional functions of neutrophils in the immune response, highlighting their role in antigen presentation and the development acquired immune responses (Ostanin et al., 2012; Chen et al., 2014; Hor et al., 2017).

Neutrophils exhibit a broad diversity of membrane receptors, whose functions include downstream activation of intracellular proteins or the transduction of networks responsible for inflammatory gene expression (Futosi et al., 2013). Toll-like Receptors (TLRs) and G-protein coupled receptors (GPCRs) are two classes of membrane receptors that are pivotal in infectious inflammatory diseases with distinct intracellular pathways. TLR4, a member of the pattern recognition receptor (PRR) family, recognizes conserved pathogen-associated molecular patterns (PAMPs) and represents the main line of defense against infections (Rosadini and Kagan, 2017). LPS binding to TLR4 activates transcription factors for the synthesis of pro-inflammatory proteins such as adhesion molecules, cytokines, and enzymes (Rosadini and Kagan, 2017). Neutrophils express several G protein-coupled receptors (GPCRs) during inflammation, such as the formyl-peptide receptor that recognizes bacteria and tissue injury products, and receptors for chemical mediators leukotriene B4 (LTB4), C5a, platelet activating factor (PAF) and some chemokines (Futosi et al., 2013). Binding of GPCR agonists activates several pathways in neutrophils to induce cell polarization leading to chemotaxis, ROS production and exocytosis of intracellular granules (Futosi et al., 2013; Dahlgren et al., 2016; Rosadini and Kagan, 2017). GPCRs activation generates phosphatidylinositol (3,4,5) triphosphate (PIP3) via PI3-kinases, and activates Rac small GTPAses, causing actin polimerization and kinase activation at the frontness of the cell. Furthermore, Rac small GTPases activate NADPH oxidase and induce ROS production in the polarized cell. This cascade of intracellular events inhibits a lipid phosphatase responsible for the degradation of PIP3 (PTEN), causing accumulation of PIP3 at the leading edge (Wang et al., 2002; Weiner, 2002; Kuiper et al., 2011).

It is well established that exacerbated neutrophil influx and activation hinders the resolution of inflammation (Paris et al., 2015; Roos et al., 2015; Chalmers et al., 2016), and that endogenous mediators modulate the development and resolution of inflammation. To address the role of TSPO in the inflammatory pathways of neutrophils, we employed different concentrations of three known TSPO ligands. Here, we aimed to elucidate TSPO’s modulation of neutrophil functions activated by distinct membrane receptors. To our knowledge, we show, for the first time, that TSPO ligands do not directly modulate the TLR4 pathway in neutrophils, but inhibit neutrophil locomotion evoked by GPCR activation by acting on cofilin and ARP2/3 functions during actin filament assembly.

## Materials and Methods

### Chemicals and Reagents

Oyster glycogen, ketamine (Dopalen), xylazine (Anasedan), TSPO ligands (Diazepam, Ro5-4864, PK-11195), dichloro-dihydro-Fluorescein diacetate (DCFH-DA), sulfanilamide, *N*-(1-nafthyl)-ethylenodiamide dihydrochlorhydrate, sodium nitrite, lipopolysaccharide isolated from *Escherichia coli* (LPS), RPMI culture medium, bovine serum albumin (BSA), Hank’s Balanced Salt Solution (HBSS), sodium thioglycolate, normal goat serum, and glycin were purchased from Sigma-Aldrich (St. Louis, MO, United States). Leukotriene B4 (LTB4) was purchased from Tocris Bioscience (Bristol, United Kingdom). Phycoerythrin (PE)-labeled mouse anti-CD62L mAB and Fluorescein isothiocyanate (FITC)-labeled CD18 mAB were purchased from BD Biosciences (San Diego, CA, United States). Anti-PBR mAB and fluorescein isothiocyanate (FITC)-labeled goat anti-rabbit were purchased from Abcam (Cambridge, MA, United States). Alexa Fluor 350 (A350)-labeled ARP2/3 mAB and FITC-labeled cofilin mAB were purchased from Bioss antibodies (Woburn, MA, United States). The actin visualization kit (phalloidin-Rhodamine) was purchased from Cytoskeleton (Denver, CO, United States). Interleukin-6 (IL-6), interleukin-10 (IL-10) and tumor necrosis factor-α (TNF-α) Elisa kits and fixation and permeabilization kits were purchased from BD Biosciences (San Diego, CA, United States). Chemotaxis plates were purchased from Neuroprobe (Leamington Spa, United Kingdom). Paraformaldehyde, ethanol, dimethylformamide (DMF), phosphoric acid and hydrogen peroxide were purchased from Labsynth (Diadema, SP, Brazil). LPS was purchased as lyophilized powder and was diluted in ultra pure water (UP water), while LTB4 was supplied pre-dissolved in anhydrous ethanol (50 μg/mL). All reagents were stored according to the product information sheet supplied by the manufacturer.

### Animals

Wild-type, male Balb/C mice weighing about 30 g (10–12 weeks old) were kept in a polypropylene box in a room in the Animal House Facility of the Chemistry Institute and Faculty of Pharmaceutical Sciences, University of São Paulo, under controlled conditions (temperature: 22–25°C; humidity: 60%; light/dark cycle: 12/12 h). The animals were provided food and water *ad libitum*, and all experiments were performed in agreement with the ethical procedures recommended for the experimental use of animals by CONCEA (*Conselho Nacional de Controle de Experimentação Animal*) and by CEUA (Ethical committee for the use of animals of the University of São Paulo; approval number CEUA n°432).

### Neutrophil and Macrophage Isolation

Oyster glycogen solution (3 mL, 1% in sterile phosphate buffer) or sodium thioglycolate (3 mL, 1% in sterile phosphate buffer) were administered to the peritoneal cavity of the mice to obtain neutrophils or macrophages, respectively. After 4 or 96 h, the animals were anesthetized (Ketamine/Xylazine, 80/8 mg/kg; s.c.) and sacrificed by carotid section to collect neutrophils or macrophages, respectively. Subsequently, the peritoneal cavity was washed with sterile cold phosphate buffer (PBS; 3 mL) to obtain neutrophil or macrophage suspensions. The suspensions were centrifuged (600 × *g*, 4°C), and the neutrophil pellets were suspended with 1 mL of Hanks balanced salt solution (HBSS) and the macrophage pellets were suspended in RPMI culture medium. Neutrophils and macrophages were counted using a haemocytometer chamber.

### Cell Treatments

Peritoneal neutrophils and macrophages were treated with Diazepam, Ro5-4864, PK-11195, 0.01% dimethylformamide (vehicle) or culture medium (R10) for 2 h at 37°C. Afterward, cells were manipulated according to the protocol of each assay.

Concentrations of 10, 100, or 1000 nM of ligands were employed to treat cells in this study. LPS was tested in concentrations of 1 or 5 μg/mL to activate neutrophils and macrophages. Neutrophils were responsive to 5 μg/mL and induced cytokines secretion, ROS production and NFkappa B nuclear translocation, as demonstrated in our previous studies (Hebeda et al., 2011, 2012). Macrophages were responsive to 1 μg/mL. Data obtained for NO2- production were (μM): Basal: 5 ± 0.3; 5 ng/mL = 20 ± 1.2; 10 ng/mL = 27 ± 1.6; 50 ng/mL = 35 ± 2.8; 1000 ng/mL = 53 ± 1.3. LTB4 was tested in a concentration of 10-10 to 10-6 M for adhesion molecule expression, chemotaxis and Arp2/3 and cofilin expressions.

### Flow Cytometry

#### TSPO Expression

Cells (1 × 10^6^) obtained as described above were incubated with inflammatory stimulus (LPS 5 μg/mL for 60 min or LTB4 100 nM for 20 min) or culture medium R10 (basal). After this period, the cells were centrifuged for 10 min (600 × *g*, 4°C), the supernatant was discarded, and the pellet was incubated with 250 μL of cytofix/Cytoperm BD solution for 30 min. The cells were blocked for non-specific binding for 30 min with glycin 0.3 M + 10% normal goat serum before incubation with a primary mAb anti-PBR (1:100 in PBS) for 30 min. The cells were then centrifuged again and incubated with FITC goat anti-IgG (1:200 in PBS) before fluorescence acquisition, which was done using a FACS Canto II cytometer (Becton-Dickinson, San Jose, CA, United States). A total of 10,000 events were analyzed, with the results expressed as median fluorescence intensity.

#### Adhesion Molecule Expression

Neutrophils were treated as mentioned above, washed with 200 μL of phosphate buffer, and further incubated with the inflammatory stimulus (LPS 5 μg/mL for 60 min or LTB4 100 nM for 20 min). Cells incubated with culture medium were employed as controls. After incubation with the stimulus, the cells were again centrifuged for 10 min (600 × *g*, 4°C). The pellets were suspended with 100 μL of monoclonal antibody anti-CD62L or anti-CD18, conjugated with PE or FITC, respectively, diluted in phosphate buffer (1:100), and further incubated for 30 min. Thereafter, the neutrophils were washed (200 μL of phosphate buffer; centrifugation for 10 min, 600 × g, 4°C), re-suspended in 200 μL of paraformaldehyde solution (2%) and analyzed by flow cytometry. A total of 10,000 events were analyzed, with results expressed as median fluorescence intensity.

#### F-Actin Expression

To assess F-actin expression, neutrophils treated as mentioned above were incubated with LTB4 (1 μM diluted with phosphate buffer) for 1.5 min. Cells were immediately fixed with 200 μL of paraformaldehyde solution (2%). Afterward, the cells were centrifuged (10 min, 600 × *g*, 4°C) and the pellets were suspended with 100 μL phalloidin conjugated with rhodamine and diluted in phosphate buffer (1:200). The cells were incubated for 30 min at room temperature and then washed with 200 μL of phosphate buffer, centrifuged (10 min, 600 × g, 4°C), suspended in 100 μL of paraformaldehyde solution (2%), and analyzed by flow cytometry. A total of 10,000 events were analyzed, with the results expressed as median fluorescence intensity.

### Cytokine Secretion

TNF-α, IL-6 and IL-10 were quantified in the supernatants of the cell cultures. Cells were treated with TSPO ligands as mentioned above and incubated with LPS (5 μg/mL) for 18 h. After this period, 100 μL of supernatant were used to quantify cytokines according to the manufacturer’s instructions for the specific ELISA kits.

### NO and ROS Quantification

#### ROS Quantification

ROS were quantified via an assay using 2′,7′-dichloro-dihydro-fluorcein diacetate (DCFH), as described by Tarpey and Fridovich (2001). Cells treated as mentioned above with TSPO ligands were incubated for 30 min with 200 μL of DCFH solution (10 μM) diluted in Ethanol (0.04%). The cells were centrifuged again and then incubated with LPS (5 μg/mL for 60 min) at 37°C. Cell fluorescence was measured (_exc_: 485 nm and _emis_: 530 nm) using a Synergy Hybrid Multi-Mode Microplate Reader (Biotek Instruments, Winooski, VT, United States). The fluorescence intensity was expressed as mean of cells’ emitted fluorescence. As a positive control, we used neutrophils and macrophages incubated with hydrogen peroxide (10 μM) for 60 min.

#### NO Quantification

NO production was indirectly assessed by nitrite quantification using the Griess assay (Tarpey and Fridovich, 2001). Cells treated with TSPO ligands were incubated with LPS (5 μg/mL) for 18 h. Then, 100 μL of culture supernatant were incubated with 100 μL of Griess reagent (1% sulfanilamide, 0.1% *N*-(1-nafthyl)-ethylenodiamide dihydrochlorhydrate diluted in phosphoric acid) for 10 min in a 96-well microplate. After this period, absorbance was read in an UV-spectrophotometer (SpectraMax 190, Molecular Devices, Sunnyvale, CA, United States) set at 540 nm. Known nitrite concentrations were used to prepare a nitrite curve, which was used to quantify the nitrite concentration present in the samples. The results were expressed as μM of nitrite present in the samples.

### Chemotaxis

To assess *in vitro* neutrophil migration, we used a previously-described assay (de Lima et al., 2012). Neutrophils treated with TSPO ligands as mentioned above were suspended in HBSS (30 μL) and transferred to the upper compartment of a ChemoTx-101-8 microplate. The bottom part of the microplate was filled with 30 μL of chemotactic agent (LTB4: 0, 0.1, 1, 10, and 100 nM, diluted in HBSS). The microplate was incubated at controlled conditions (37°C, 5% CO_2_) for 2 h. Cell numbers in the bottom compartments were obtained using a haemocytometer and an optical microscope.

### Confocal Microscopy

Actin filaments (F-actin), Actin Related Protein 2 and 3 (ARP2/3) and cofilin expression were assessed by confocal microscopy using a Zeiss LSM 780 NLO Incell Analyser 2200 GE (Oberkochen, Stuttgart, Germany). Cells treated with TSPO ligands as mentioned above were treated with 200 μL of LTB4 (1 μM diluted in phosphate buffer) for 1.5 min (90 s). The cells were then immediately fixed and permeabilized, as described by the kit manufacturer’s instructions. Thereafter, 1:200 diluted conjugated antibody anti-ARP2/3, anti-Cofilin or phalloidin-Rhodamine (100 μL) was added to the cells. Cells were incubated for 30 min, washed, centrifuged (10 min, 600 × *g*, 4°C) and analysed by confocal microscopy. Fluorescence intensity from 10 to 50 cells per experimental group was quantified using Image J software^1^ and results were expressed as mean fluorescence intensity per group.

### ARP2/3 Quantitative Protein Expression

Actin related protein 2/3 (ARP2/3) quantitative protein expression in neutrophils was assessed by western blot. Neutrophils (1 × 10^7^ cells) were obtained and treated as mentioned above and further stimulated with LTB4 (1 μM diluted with phosphate buffer pH 7,0) for 1.5 min. Cells were immediately centrifuged thereafter (10 min, 600 × *g*, 4°C), then pellets were suspended with lysing buffer (25 mM Tris-HCl pH 7,2; 150 mM NaCl; 5 mM MgCl2; 1% NP-40; 5% glycerol, protease and phosphatase inhibitors 1:300, v/v; Thermo Scientific, Rockford, IL, United State), and incubated for 5 min on ice. Samples were centrifuged again (16000 × *g*, 15 min, 4°C) and total protein were quantified by BCA assay (Smith et al., 1985) using the protein extract obtained. Proteins (60 μg) were separated by a polyacrylamide gel (10%) and electrophoretically transferred to a nitrocellulose membrane. After non-specific sites blocking using TBS-T (tris-buffered saline – Tween 20) supplemented with BSA (5%) for 1 h at room temperature, membranes were incubated overnight at 4°C with a primary rabbit polyclonal anti-Arp2/3 antibody (1:100) (Abcam, Cambridge, MA, United States). Primary monoclonal mouse Anti-vinculin antibody (Sigma–Aldrich, St. Louis, MO, United States) was used as control for protein concentration applied to the gel. Membranes were washed three times with TBS-T and incubated for 2 h with a horseradish peroxidade-conjugated goat anti-rabbit secondary antibody (Abcam, Cambridge, MA, United States) diluted in TBS-T supplemented with BSA (5%). Immunoreactive bands detection was performed by a chemiluminescence assay using a ECL revelation kit (Enhanced chemiluminescent substrate, Thermo Scientific, Rockford, IL, United States) and a chemiluminescence system (UVITEC Cambridge, United Kingdom). Quantitative immunoreactive bands analysis were performed using ImageJ software^1^ and results were expressed as mean fluorescence intensity per group.

### Statistics

Data are presented as mean ± SEM. Comparisons between the two groups were made using *t*-test (Student). When more than two groups were compared, we used ANOVA followed by Dunnet test or multicomparison test. Differences were considered significant when the *P*-value was below than 0.05 (*P* < 0.05). Prism GraphPad v 5.0 (GraphPad Software, Inc., La Jolla, CA, United States) was employed.

## Results

**FIGURE 1:**
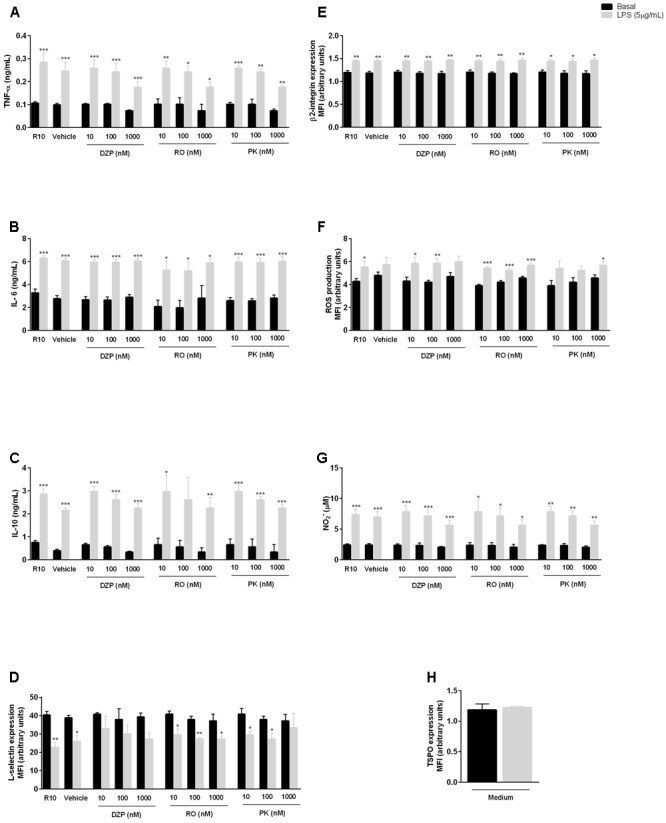
Role of TSPO ligands in LPS effects on neutrophils. Mice peritoneal neutrophils were incubated with TSPO ligands (Diazepam [DZP], Ro5-4864 [RO] or PK11195 [PK]) for 2 h and further incubated with culture medium (basal) or LPS for 18 h to quantify TNF-α **(A)**, IL-6 **(B)**, IL-10 **(C)**, and NO **(G)**, or for 1 h to determine the expression of adhesion molecules L-selectin **(D)** and β2 integrin **(E)**, ROS production **(F)**, and TSPO expression **(H)**. The data are expressed as mean ± SEM of cells collected from 4 animals in each group. ^∗^*P* < 0.05; ^∗∗^*P* < 0.01; ^∗∗∗^*P* < 0.001 vs. basal values. The data were statistically analyzed by ANOVA followed by Dunnet test.

Activation of neutrophils using LPS enhanced the secretion of TNF-α, IL-6 and IL-10 by neutrophils (**Figures 1A–C**), while pre-treatment with diazepam, PK11193 or Ro5-4864 did not alter basal or LPS-induced cytokine secretion (**Figures 1A–C**). Moreover, activation of leukocytes by LPS caused L-selectin shedding from the neutrophil membrane and increased β2 integrin expression on the cell surface, effects that were not modified by pre-incubation with TSPO ligands (**Figures 1D,E**). We also observed that LPS induced ROS and RNS production by activating the oxidative burst and the production of nitric oxide (NO), respectively, and pre-treatment with diazepam, PK11193 or Ro5-4864 did not alter ROS and RNS production (**Figures 1F,G**). Finally, we observed that TSPO expression was not enhanced by LPS treatment (**Figure 1H**).

**FIGURE 2:**
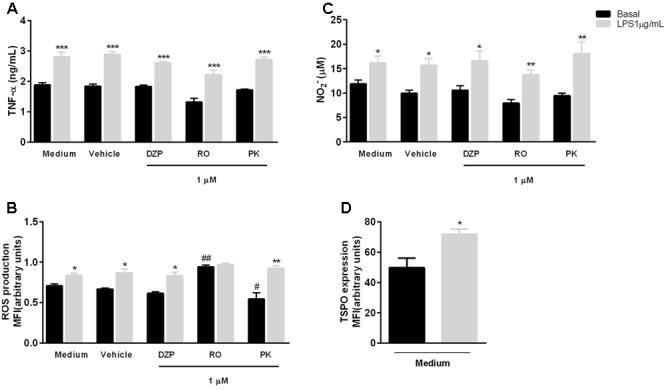
Role of TSPO ligands in LPS effects on macrophages. Mice peritoneal macrophages were incubated with TSPO ligands (Diazepam [DZP], Ro5-4864 [RO] or PK11195 [PK]) for 2 h and further incubated with culture medium (basal) or LPS for 18 h to quantify TNF-α **(A)** and NO **(C)**, or for 1 h to determine ROS production **(B)** and TSPO expression **(D)**. The data are expressed as mean ± SEM of cells collected from 4 animals in each group. ^∗^*P* < 0.05; ^∗∗^*P* < 0.01; ^∗∗∗^*P* < 0.001 vs. basal values, and ^#^*P* < 0.05 vs. respective value in RO group; ^##^*P* < 0.01 vs. respective value in R10 and vehicle groups. The data were statistically analyzed by ANOVA followed by Dunnet test.

The same profile of TSPO ligand action on neutrophils was observed in macrophages after LPS stimulation (1 μg/ml). Macrophages were more responsive to LPS than neutrophils, and the concentration of LPS employed to activate them was lower and in accordance to the literature (Rosenfeld et al., 2006; Ko et al., 2017). LPS treatment enhanced TNF-α secretion, NO production and ROS expression, and pre-treatment with diazepam, PK11193 or Ro5-4864 did not modify these effects (**Figures 2A–C**). It is interesting to mention that RO and PK treatments modified the basal production of ROS, nevertheless RO enhanced and PK reduced ROS production. Increased ROS production after RO treatment was previously reported in astrocytes and microglia (Jayakumar et al., 2002; Choi et al., 2011). It was suggested that NADPH-oxidase activation may be involved in this effect (Choi et al., 2011). One hour after LPS incubation, enhanced TSPO expression was observed in macrophages (**Figure 2D**).

**FIGURE 3:**
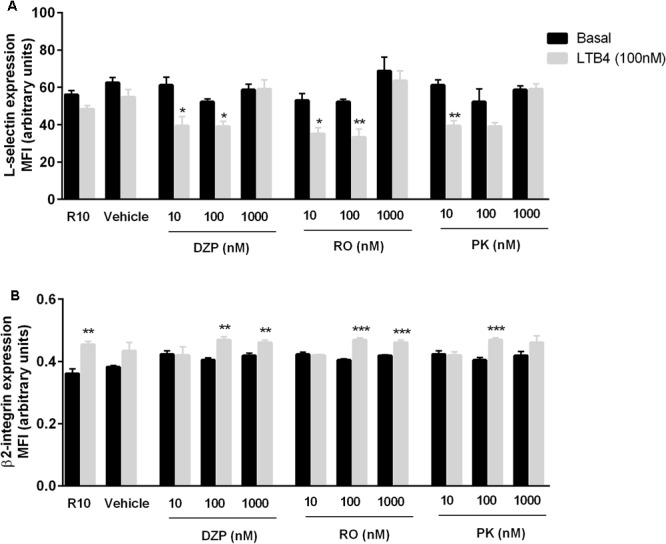
Role of TSPO ligands in LTB4-induced expression of adhesion molecules by neutrophils. Mice peritoneal neutrophils were incubated with TSPO ligands (Diazepam [DZP], Ro5-4864 [RO] or PK11195 [PK]) for 2 h and further incubated with culture medium (basal) or LTB4 100 nM for 20 min to quantify L-selectin **(A)** and β2 integrin **(B)** expression. The data are expressed as mean ± SEM of cells collected from 4 animals in each group. ^∗^*P* < 0.05; ^∗∗^*P* < 0.01; ^∗∗∗^*P* < 0.001 vs. basal values. The data were statistically analyzed by ANOVA followed by Dunnet test.

Our previous data showed that TSPO ligands alter the response of neutrophils to *N*-formylmethionyl-leucyl-phenylalanine (fMLP; (de Lima et al., 2012)), a protein derived from bacteria and a GPCR ligand. Therefore, to test whether TSPO activation interferes with GPCR activation by chemical mediators, we activated neutrophils with LTB4. LTB4 cleaved L-selectin from the neutrophil membrane, an effect that was blocked by the highest concentrations of all TSPO ligands (**Figure 3A**). Moreover, LTB4 activation induced β2 integrin expression, an effect that was only blocked by the lowest concentrations of diazepam, Pk11193 and Ro5-4864 (**Figure 3B**).

**FIGURE 4:**
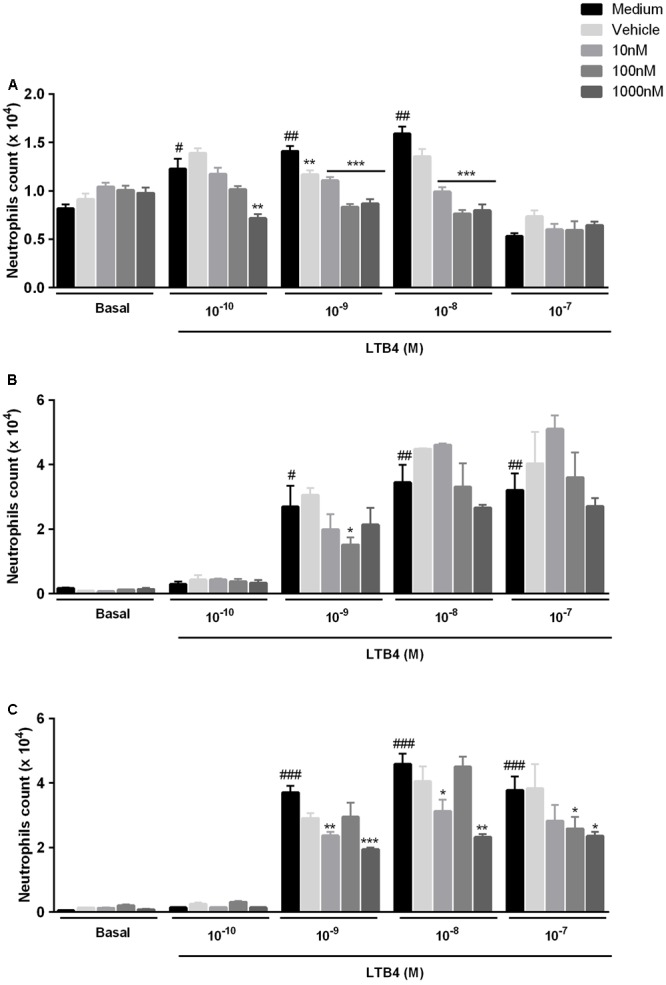
Role of TSPO ligands in LTB4-induced neutrophil chemotaxis. Mice peritoneal neutrophils were incubated with TSPO ligands (Diazepam **[A]**, Ro5-4864 **[B]** or PK11195 **[C]**) for 2 h and further incubated with culture medium (basal) or LTB4 (0.1–100 nM) for 2 h to quantify the number of migrated neutrophils. The data are expressed as mean ± SEM of cells collected from 6 animals in each group. ^#^*P* < 0.05 ^##^*P* < 0.01 vs. medium in basal group; ^∗∗^*P* < 0.01; ^∗∗∗^*P* < 0.001 vs. respective medium values. The data were statistically analyzed by ANOVA followed by Dunnet test.

**FIGURE 5:**
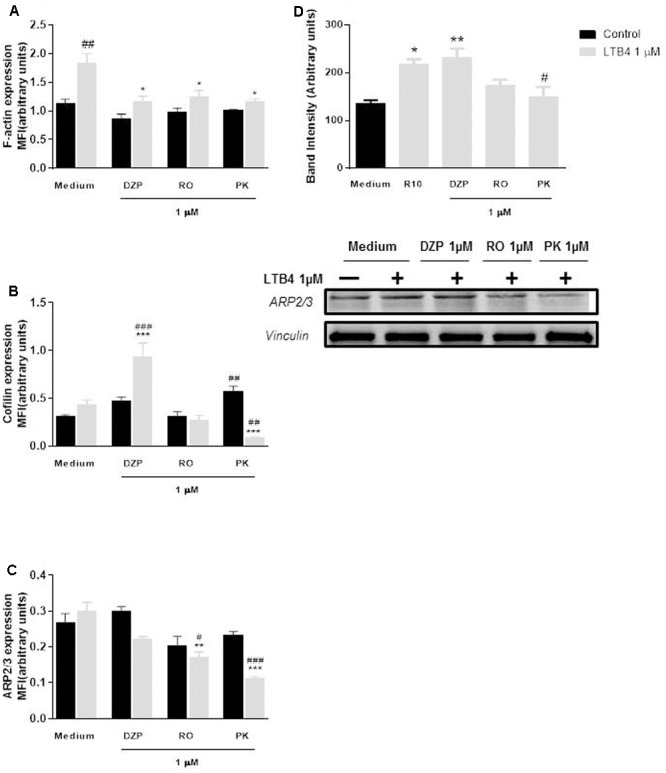
Role of TSPO ligands in LTB4-induced actin polymerization and cofilin and ArP2/3 expression by neutrophils. Mice peritoneal neutrophils were incubated with TSPO ligands (Diazepam [DZP], Ro5-4864 [RO] or PK11195 [PK]) for 2 h and further incubated with culture medium (basal) or LTB4 1000 nM for 1.5 min (90 s) to quantify actin polymerization **(A)**, expression of cofilin **(B)**, expression of ARP2/3 by confocal microscopy **(C)** and by western blot **(D)**. The data are expressed as mean ± SEM. cells collected from 4 animals in each group for confocal microscopy and 4 western blot analysis using a pool of neutrophils obtained from 8 animals in each analysis. ^##^*P* < 0.01; ^###^*P* < 0.001 vs. respective medium; ^∗^*P* < 0.05; ^∗∗^*P* < 0.01; ^∗∗∗^*P* < 0.001 vs. LTB medium group; vs. respective basal value. ^∗^*P* < 0.05; ^∗∗^*P* < 0.01 vs. medium control group and ^#^*P* < 0.05 vs. medium stimulated group (For western blot analysis). The data were statistically analyzed by ANOVA followed by Dunnet test.

**FIGURE 6:**
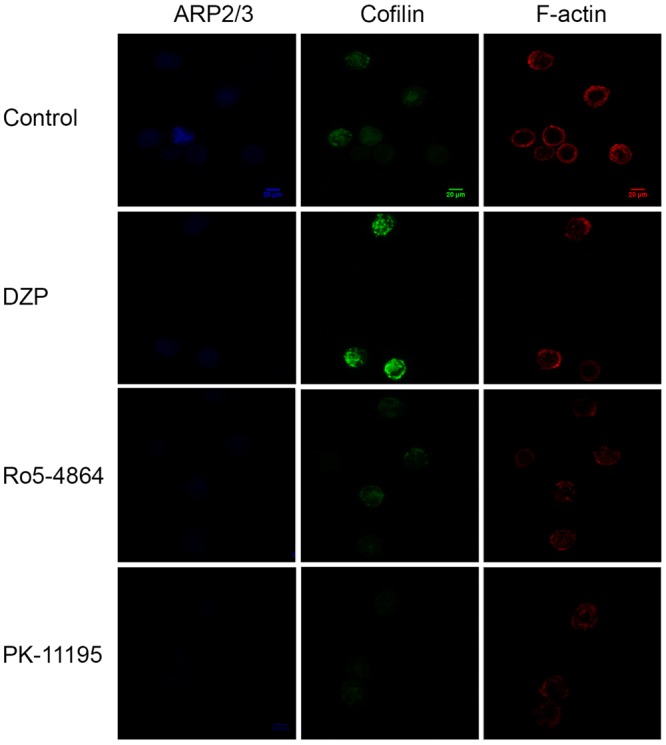
Representative images from confocal microscopy. Green represents cofilin expression, red represents actin polymerization and blue represents Arp2/3 expression.

LTB4 induced neutrophil chemotaxis in a concentration dependent manner, and this effect was blocked by all ligands of TSPO (**Figure 4**). Chemotaxis is dependent on polymerization of actin filaments. Here, we found that LTB4 induced actin filament assembly, and that this effect was significantly impaired by pre-treatment with the three TSPO ligands (**Figure 5A**). Further experiments showed that the TSPO ligands influence neutrophil chemotaxis and actin polymerization via different mechanisms. Treatment with Diazepam enhanced cofilin expression and did not alter ARP2/3 expression; Ro5-4864 treatment did not modify cofilin or ARP-2/3 expression, and PK11195 treatment markedly reduced both cofilin and ARP-2/3 expression (**Figures 5B–D**). Images obtained by confocal microscopy are shown in **Figure 6**.

## Discussion

To our knowledge, we show, for the first time, the specific actions of TSPO ligands in the inflammatory pathways of peripheral phagocytes. TSPO ligands inhibited the actin filament assembly and neutrophil locomotion elicited by LTB4-induced GPCR activation, but did not directly inhibit the TLR4 pathways activated by LPS in neutrophils and macrophages.

Based on the evidence that the effects of TSPO ligands depend on the cell type, site of binding on the receptor, and chemical structure and concentration of ligand (Caballero et al., 2014; Costa et al., 2015; Selvaraj and Tu, 2016), we employed a wide range of concentrations of three prototypical TSPO ligands, from nanomolar to micromolar, the latter being considered very high and toxic. It is important to mention that we did not observe any alterations of cell viability, as measured by trypan blue eclusion test (data not shown).

Upon LPS activation, TLR4 rapidly induces the assembly of the adaptor proteins MyD88 (myeloid differentiation primary response gene 88) and TIRAP, as well as several serine threonine kinases of the interleukin-1 receptor-associated kinase (IRAK) family. This subcellular site promotes nuclear factor kB (NF-kB) and activating protein-1 (AP-1) activation, leading to inflammatory gene expression. Moreover, through MyD88 activation, LPS/TLR4 activates c-Src/NADPH oxidase to generate ROS. Subsequently, TLR4 is internalized into the endosomes, where it promotes interferon regulatory factor 3(IRF3)-dependent type-I interferon (IFN) secretion through the adaptor proteins TRAM and TRIF (Rosadini and Kagan, 2017). LPS has been employed as an inflammatory agent to investigate TSPO actions, as TSPO is markedly expressed in different cells following systemic or local injection of LPS, such as in CNS, liver, and lungs (Veiga et al., 2007; Choi et al., 2011; Hatori et al., 2012; Karlstetter et al., 2014; Sandiego et al., 2015; Wang et al., 2016; Zhang et al., 2016) and in cultured microglia (Choi et al., 2011; Shao et al., 2013; Karlstetter et al., 2014; Pihlaja et al., 2015). Moreover, it has been shown that TSPO ligands alter the inflammatory process evoked by LPS, with TSPO ligand vinpocetine inhibiting NO production and IL-1 β, IL-6, and TNF-α secretion by LPS-stimulated microglia (Shao et al., 2013), and the TSPO ligand XBD173 suppressing the transcription of pro-inflammatory genes chemokine (C-C motif) ligand 2 (CCL2), IL-6 and inducible NO-synthase (iNOS) by LPS-activated microglia (Karlstetter et al., 2014). Conversely, Ro5-4864 and PK11195 treatments had no effect on pro-inflammatory gene expression evoked by LPS in microglia, while Ro5-4864 treatment enhanced LPS-induced release of IL-1β by microglia (Choi et al., 2011). It was therefore surprising that TSPO expression was not increased in LPS-activated neutrophils, and that none of the TSPO ligands employed affected the inflammatory pathways induced by TLR4 activation. We hypothesized that neutrophils could have different intracellular responses to TSPO ligands following LPS activation than other phagocytes, such as microglia and macrophages. Hence, we investigated the effects of TSPO ligands in peritoneal macrophages activated by LPS. TSPO levels were augmented by LPS treatment, but like neutrophils, treatment with TSPO ligands did not alter the effects of LPS on macrophages. Together, our data show that diazepam, Ro5-4864 and PK11195 do not alter the effects of LPS on peripheral phagocytes in a way that is different to that observed in microglia (Choi et al., 2011; Karlstetter et al., 2014; Pihlaja et al., 2015; Shao et al., 2013). Therefore, we propose that TSPO’s role in systemic and local LPS-induced inflammation at peripheral sites may be indirect, via interference with pathways activated by mediators secreted following LPS administration. Indeed, it has been shown that TSPO overexpression or TSPO ligands reduce TNFα’s inflammatory effects on endothelial and gastric epithelial cells (Joo et al., 2012; Issop et al., 2016).

We have previously shown that TSPO ligands alter the GPCR pathways of neutrophils when activated by fMLP, a bacteria-derived peptide. Local Ro5-4864 treatment abrogated fMLP-induced leukocyte-endothelial interactions in the mesenteric postcapillary venules of rats, while we saw opposite effects of *in vitro* Ro5-4864 or PK11195 treatment on chemotaxis and enhancement of intracellular calcium [iCa(+2)]. We also uncovered an allosteric agonist/antagonist relationship of TSPO activation in neutrophils, as the effects of Ro5-4864 on fMLP-stimulated neutrophils were reverted by simultaneous treatment with PK11195 (de Lima et al., 2012).

As GPCRs represent the largest family of integral membrane receptors and contribute to all known physiological processes in mammals (Farran, 2017), we investigated whether TSPO ligands could alter the inflammatory GPCR pathway stimulated by an endogenous mediator. LTB4 is produced by metabolization of arachidonic acid by 5-lipoxygenase, and interacts with two distinct GPCRs on the cell surface, named LTB4 receptor type 1 (BLT1) and BLT2 (Bäck et al., 2014; Le Bel et al., 2014; Mashima and Okuyama, 2015). BLT1 shows higher affinity for LTB4 than BLT2, is highly expressed on neutrophils, and mediates the focal adhesion and chemotaxis evoked by LTB4 (Yokomizo et al., 1997, 2000; Monteiro et al., 2011; Batra et al., 2012; Futosi et al., 2013; Yokomizo, 2015).

We show here that LTB4 stimulation evokes L-selectin shedding from the cell membrane and increases the expression of CD18 on the cell surface. It has been well established that this profile of adhesion molecule expression is pivotal to the proper interaction between neutrophils to vessel wall, and their subsequent migration into inflammatory sites (Mitroulis et al., 2015). Treatment with TSPO ligands inhibited the respective downregulation and upreguation of L-selectin and β2 integrin expression by LTB4, indicating that TSPO ligands interfere with neutrophil interaction with the vessel wall in inflamed tissues. Inhibition of L-selectin shedding was induced by the highest concentration of ligands, while blockade of β2 integrin overexpression was evoked by the lowest concentration of TSPO ligands. Cleavage of L-selectin from the cell membrane is mediated by metalloproteases such as disintegrin and metalloproteinase 17/Tumor necrosis factor-α-converting enzyme (ADAM-17/TACE), and the expression of β2 integrin is dependent on the mobilization of storage pools in neutrophil secretory granules or on gene transcription (Ramadass and Catz, 2016). The ability of TSPO ligands to affect the expression or activity of membrane ADAM-17/TACE, or mobilization and/or synthesis of β2 integrin should be further evaluated.

Although it has been previously shown that TSPO ligands influence neutrophil locomotion, the data are controversial and point to different mechanisms of action. For example, Cosentino et al. (2000) found that Ro5-4864 treatment did not enhance human neutrophil migration, while our data demonstrate that Ro5-4864 and PK11195 respectively inhibit and augment fMLP-induced neutrophil chemotaxis (de Lima et al., 2012). Here, the three TSPO ligands employed inhibited LTB4-activated neutrophil locomotion and impaired actin assembly. The intracellular pathway of neutrophil locomotion is highly complex, and has not been fully elucidated. Upon BLT activation, the G protein dissociates into the Gα and Gβ subunits, and both activate downstream effectors of neutrophil chemotaxis by inducing the constant generation of a meshwork of F-actin, which pushes the cell forward in directional cell migration (Neptune and Bourne, 1997; Li et al., 2000; Xu et al., 2003). GPCR signaling via Rho small GTPases stimulates the activity of the Arp2/3 complex at the leading edge of neutrophils to initiate the formation of new branches of actin filaments (Servant et al., 2000; Welch and Mullins, 2002; Li et al., 2005; Gan et al., 2012). Moreover, Gβγ subunits activate the PLCβ/phosphoinositide 3-kinase gamma□(PI3Kgamma-GSK3) pathway to trigger downstream expression of cofilin, a low-molecular-weight actin regulatory protein that is localized at the leading edge and is involved in actin remodeling (Tang et al., 2011). Our data clearly show that diazepam treatment enhanced, and Ro5-4864 and PK11195 treatment reduced, cofilin expression in LTB4-activated neutrophils, with a more marked effect of PK11195. Moreover, levels of ARP2/3 were reduced by both Ro5-4864 and PK11195 treatment, but not by diazepam treatment. Therefore, our data clearly show that different TSPO ligands act via distinct molecular mechanisms to influence neutrophil locomotion, and we suggest that TSPO activation may be a relevant tool for controlling neutrophil infiltration of inflammatory sites.

Together, the data presented herein further elucidate the mechanisms of TSPO ligands by showing, for the first time, the inability of TSPO ligands to directly interfere with TLR-4 pathways in neutrophils. Moreover, the data corroborate the pivotal effects of TSPO ligands on GPCR-activated pathways, with distinct inhibition of GPCR-induced neutrophil locomotion. Thus, TSPO plays an important role in modulating the inflammatory disorders elicited by GPCR activation, and our study opens new insights into TSPO’s role in GPCR activation in other biological processes.

## Author Contributions

LK, EB, and CD performed experiments and analysed the data. CN and SS contributed to confocal experiments. SS contributed to the writing of the manuscript. SF was the leader of the project, and contributed to the writing of the manuscript.

## Conflict of Interest Statement

The authors declare that the research was conducted in the absence of any commercial or financial relationships that could be construed as a potential conflict of interest. The reviewer PB and handling Editor declared their shared affiliation.

## Footnotes

1 https://imagej.nih.gov/ij/
